# Continuous Auditing and Continuous Certification of Cloud Services in MEDINA – Security Auditor’s View

**DOI:** 10.12688/openreseurope.16703.2

**Published:** 2024-05-01

**Authors:** Tatu Suhonen, Cristina Martínez

**Affiliations:** 1 Nixu Certification Ltd, ESPOO, 02150, Finland; 2 Fundacion Tecnalia Research & Innovation, San Sebastián, Basque Country, E-20009, Spain

**Keywords:** EUCS, Continuous Auditing, Continuous Certification

## Abstract

This paper discusses views on continuous auditing and continuous certification of cloud services in the context of the MEDINA EU project and the security auditing industry. Based on an introduction of MEDINA, the notions of continuous auditing and continuous certification are introduced from a security auditor’s perspective to discuss the opportunities and challenges related to these topics. The paper also discusses further actions beyond this project in order to provide feedback on how continuous auditing and certification can be developed and introduced to the market.

## Disclaimer

The views expressed in this article are those of the authors. Publication in Open Research Europe does not imply endorsement of the European Commission.

## Introduction

One of the recognized reasons for the still limited adoption of Cloud Computing in the EU is the customers’ perceived lack of security and transparency in this technology ^1, 2^. Cloud Service Providers (CSPs) usually rely on security certifications as a means to improve transparency and trustworthiness. However, European CSPs still face multiple challenges for certifying their services (e.g., fragmentation in the certification market, and lack of mutual recognition of certification schemes adding workload related to maintaining certificates). In this context, the EU Cybersecurity Act (EU CSA, approved in June 2019 ^3^) proposes improving customers’ trust in the European ICT market through a set of EU-wide certification schemes. One of those schemes, the European Cybersecurity Certification Scheme for Cloud Service (EUCS) ^4^ is being developed by the European Union Agency for Cybersecurity (ENISA). For a selected set of high-level assurance requirements, the EUCS proposes the following notion of continuous (automated) monitoring: the requirements related to continuous monitoring typically mention “automated monitoring” or “automatically monitor” in their text. The intended meaning of “monitor automatically” is:

1. Gather data to analyse some aspects of the activity being monitored at discrete intervals at a sufficient frequency.
2. Compare the gathered data to a reference or otherwise determine conformity to specified requirements in the EUCS scheme.
3. Report deviations to subject matter experts who can analyse the deviations in a timely manner.
4. If the deviation indicates a nonconformity, then initiate a process for fixing the nonconformity.
5. If the non-conformity is major, notify the Conformance Assessment Body (CAB) of the issue, analysis, and planned resolution.

These requirements stop short of requiring any notion of continuous auditing, because technologies have not reached an adequate level of maturity. Nevertheless, the introduction of continuous auditing, at least for high-level assurance requirements, remains a mid- or long-term objective, and the introduction of automated monitoring requirements in at least some areas is a first step in that direction, which can be met with the technology available today. The EUCS notion of continuous monitoring conveys important technological and organizational challenges for stakeholders, which need to be carefully analysed and understood by all relevant stakeholders in order to benefit the adoption of this new certification scheme.

This paper focuses on discussing the notion of continuous auditing and continuous certification for cloud services from the perspective of an auditor. The topic has already been addressed in different contexts for some years from a general auditing perspective, but more in research than in practical implementations. For example in research articles ^5– 7^, but also in other certification schemes, such as the CSA STAR, which includes a continuous assessment aspect in level 3 assessments. In terms of already existing tools, many hyperscalers and technology providers offer security compliance monitoring tools, but they might not provide an auditor interface, since their preferred use case is as an internal monitoring tool.

The observations and future recommendations provided in this paper are based on the innovations of the MEDINA project ^8^ and the expertise of a Conformance Assessment Body (Nixu) working as one of the partners in the project as subject matter experts.

## MEDINA framework

The scope of the continuous certification in MEDINA is a set of the assurance level ‘high’ security controls defined in the EUCS scheme that are of assurance level high and include the wording “automatically monitor” or variations thereof. Both technical and organizational objectives can be continuously monitored in MEDINA, although the periodicity defined for the latter is much higher than for the former.

This section provides a simplified and brief overview of the MEDINA framework. For a broader understanding of the MEDINA framework and its components, it is recommended to read the paper “An architecture proposal for the MEDINA framework” ^9^. MEDINA is based on four main pillars that make the project relevant to the notion of continuous and automated monitoring for the security certification of cloud services:

1. **Metrics repository**: The EUCS provides a set of security requirements that shall be leveraged to certify cloud services. The lack of defined “compliance metrics” for assessing EUCS requirements could be a problem for Cloud Service Providers (CSP) and Conformance Assessment Bodies (CAB), which may have to leverage their own customised metrics for automatic application/assessment. To address this, the MEDINA framework includes a Catalogue of controls and metrics associated with the EUCS requirements, covering security topics such as information security organization, asset management, operational security, incident management and business continuity. More than 150 metrics have been defined, based on literature, other European projects (EU FP7 CUMULUS, A4Cloud and SPECS) and the work of MEDINA partners. Metrics produce quantifiable information to be compared with a target value; are collected on a regular basis for continuous monitoring; and have been described following the same structure, which includes the defined data type, data range, target value, operator and interval ^10^. An example of a metric that can be monitored continuously is the "MalwareProtectionEnabled" metric, a technical metric that supports the achievement of the OPS-05.3H operational security requirement by checking if an antivirus system is enabled on a virtual machine resource. The data range defined for this metric is {true, false}, its target value is "True", the operator used to check compliance is "==" and the interval to do so is 1 hour.
2. **Risk-based approach for security controls**: The MEDINA framework includes a risk-based methodology that relies on a tool for the analysis of EUCS requirements, ensuring that compliance with these requirements is actually relevant for the CSP, depending on its risk appetite. The tool evaluates the non-conformities and determines whether a non-conformity is major -leading to revocation of the certificate-, or the deviation is minor -and therefore the certificate should be maintained ^11^.
3. **Certification language**: EUCS requirements are defined in natural language, so it is necessary to "translate" them into a machine-readable representation that facilitates the extraction of metrics. To this end, the MEDINA framework includes tools that make use of NLP (Natural Language Processing) techniques to recommend metrics for the evaluation of a given requirement, as well as for the extraction of the values of organisational metrics within normative documents ^12^.
4. **Evidence collection and continuous audit**: In order to achieve continuous audit-based certification of cloud services, it is necessary to collect real technical evidence related to automated monitoring. To this end, the MEDINA framework includes tools and methodologies that manage the collection of evidence at both code and service level, thus supporting CSPs to apply for a high-assurance EUCS certificate. Digital evidence is continuously monitored and evaluated by MEDINA, and Blockchain techniques are used to implement accountable monitoring ^13^.

**Figure 1.**
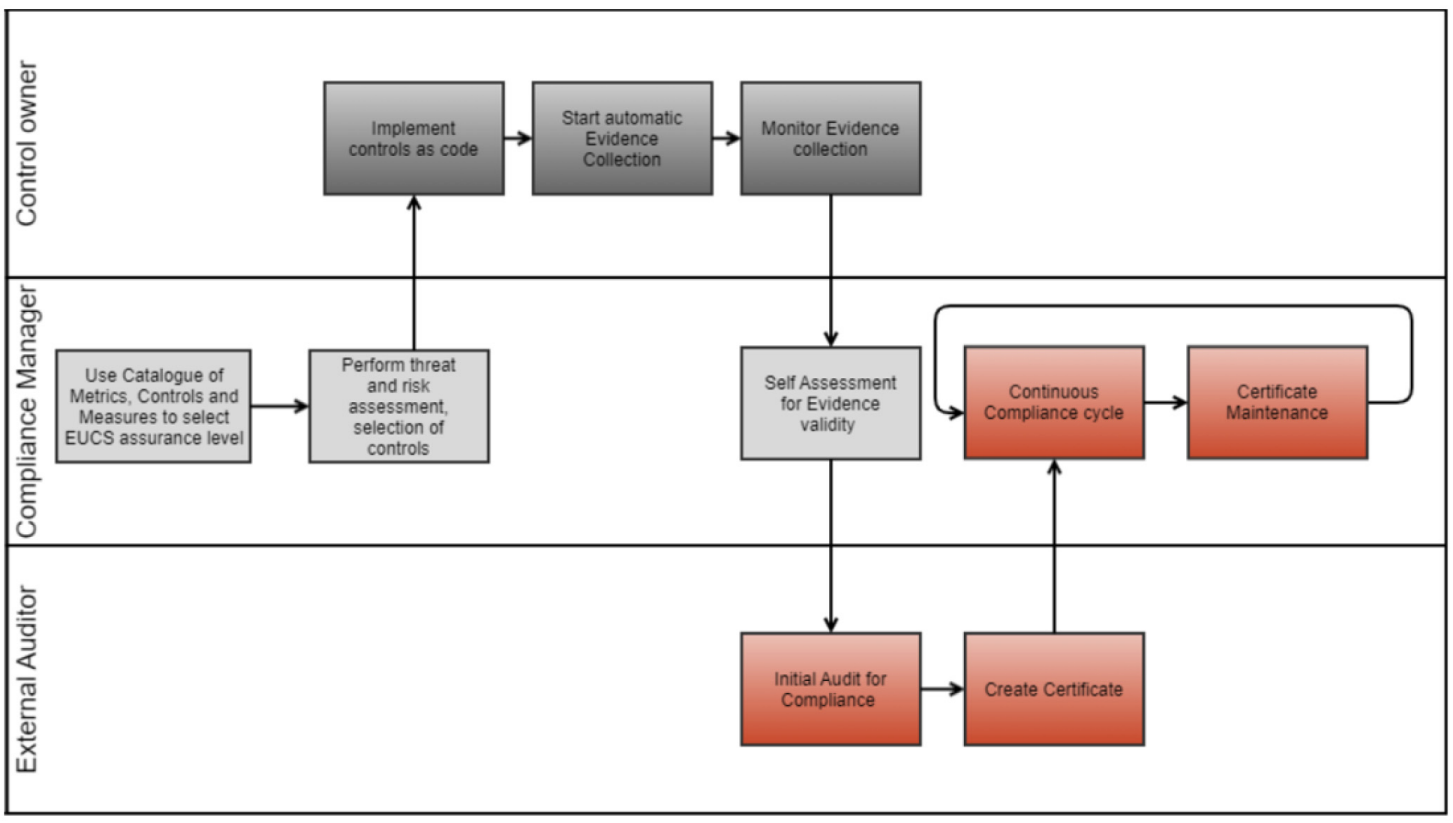
High-level MEDINA workflow.

Figure 1 shows the high-level workflow of the MEDINA framework. Users of the solution, i.e., control owners, compliance managers and external auditors, have their own swimming lane in the workflow and time flows from left to right. The components involved in each phase of the workflow are covered in more detail in 9. In addition, MEDINA deliverables ^1^ cover in detail each component and each phase of the framework processes. This paper focuses on those phases where the auditor is involved, i.e., the boxes marked in red in Figure 1.

## MEDINA for auditors

In this section we discuss the effects of MEDINA to information security auditing. The analysis includes discussing changes to audit processes and audit methods in MEDINA, not forgetting the higher-level strategic discussion related to changes in the auditing industry driven by the need for continuous auditing.

### From point-in-time audits to continuous auditing

MEDINA is offering a solution to one of the challenges in the current audit practice, which is the uncertainty of conformity between audits due to a lack of auditor presence in the auditee’s environment between audits ^14^. Why is this a problem? When an auditee suffers a breach or deliberately (or by accident) acts against the rules of certification, it causes a reputation and brand impact to the CAB. Of course, it is the CAB’s interest to do their job with utmost professionalism, but anything can happen in-between audits. Perhaps a key employee of the auditee resigns, and security gradually starts to fail.

Most certification schemes follow an approach in which an onsite audit must be completed successfully before a certificate is granted. The certificate has a validity period, for example three years after which it must be renewed with another thorough audit, a recertification audit. Currently, to tackle the uncertainty with the auditee conforming to the requirements during the certificate’s validity period, some certification schemes, if not requiring an annual re-audit, require mandatory smaller scale surveillance audits, typically annually. Surveillance audits aim to ensure that any changes in the certification scope can be assessed and most importantly aim to ensure that the auditee’s processes and actions still meet the criteria of certification. For example, the standard ISO/IEC 17021-1 ^15^, which is applied to CABs providing ISO/IEC 27001 audits, enforces the surveillance audits in order to maintain confidence that the auditee still fulfils the requirements after the initial audit.

The challenge in this approach has always been that the audit is always a representation of the auditee’s current state during one point in time, and therefore the name *point-in-time audit*. There are limited ways to ensure that the auditee is at least maintaining the same level of security between audits, most importantly after the initial audit has been finished and the certificate has been granted. In some cases, the auditee may consider the certification audit more as an annual project rather than as a continuous and integral part of daily work and is thus focusing majority of their efforts just prior to the audits. Certification (and security in general) is not a sprint; it is a continuous process. Ultimately an organisation should aim to be proactive instead of reactive and this is where continuous auditing may help. The relatively long period of inactivity between certification activities creates a certain trust factor in play. The auditor cannot always be present at the auditee and thus the opportunities to review the conformity status of an auditee are almost only limited to the surveillance audits, or if a complaint is filed against a certificate by a third party. Furthermore, the auditor can never achieve a 100% certainty in the audit results as audits are usually based on sampling.

**Figure 2.**
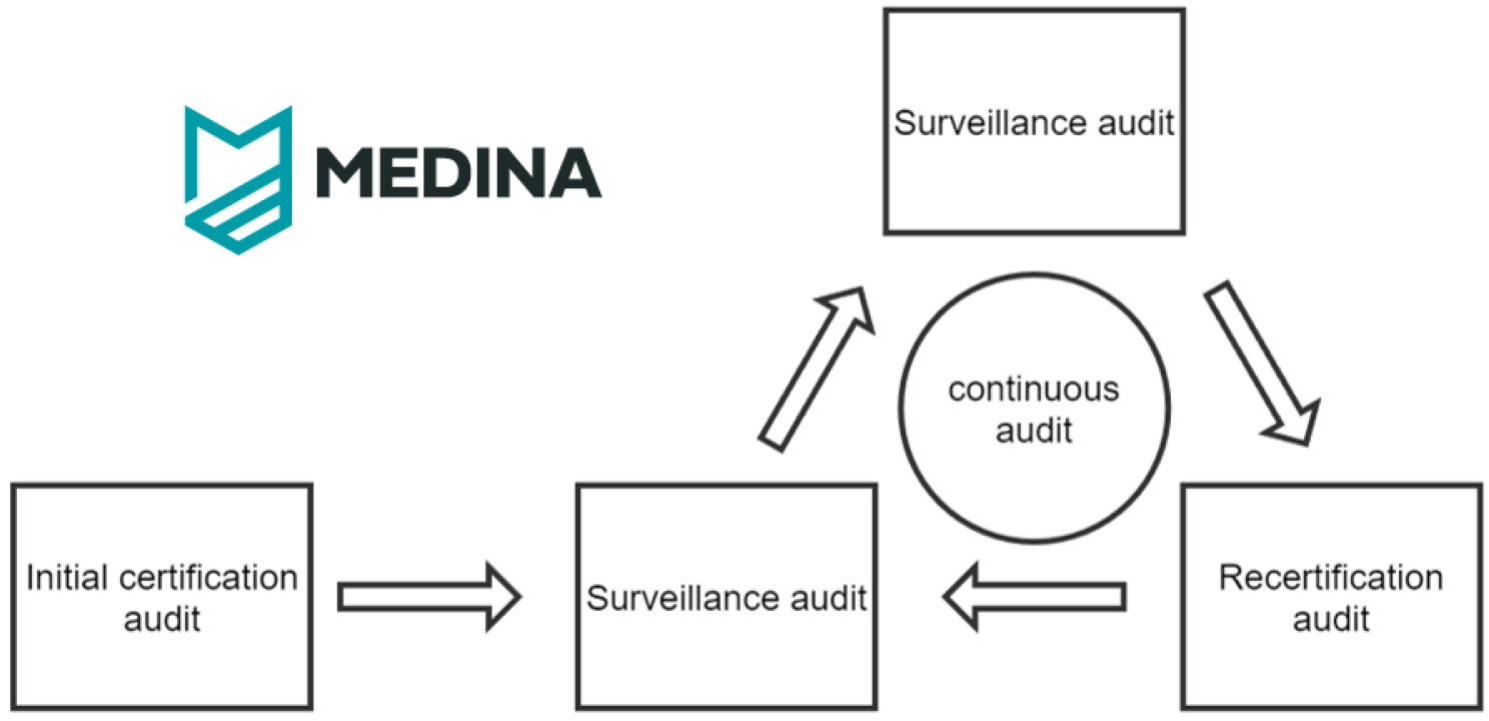
Simplified certification life-cycle.

By introducing continuous auditing, the uncertainty of compliance status between onsite audits can be lowered. Continuous auditing is achieved with MEDINA framework components by mapping the requirements to measurable metrics which in turn can be used to collect and assess evidence ^16^. This evidence can be then evaluated by the auditor in each onsite audit throughout the audit cycle. A simplified illustration of the certification cycle is presented in Figure 2.

Continuous auditing offers great opportunities both for the auditee and for the auditor. While the auditee can increase their general security awareness and enhance their security posture by implementing continuous monitoring and auditing capabilities and eventually increase customer trust, the auditor gets more assurance of the auditee’s compliance throughout the certification cycle and has more extensive audit methods in use by utilizing automated tools to assist in gathering and storing evidence. For example, when doing an onsite audit, the auditor has only a limited time to analyse and audit a sample of cloud resources, even with the help of tools.

With continuous auditing the sample size can be extended significantly, and focus can be put on finding and analysing any anomalies in the audit results since the evaluation has been done automatically for the auditor throughout the audit period. By extending the continuous auditing to continuous certification, the auditor can even further extend the usability of MEDINA by integrating the certificate life cycle management on top of continuous auditing ^17^. This allows the auditor to get notified of non-conformities, and react to them accordingly, for example, by updating the certificate status to suspended in case of a nonconformity. Continuous certification is studied in more detail later in this paper.

It is important to notice that the implementation of automated tools does not necessarily reduce the workload of an auditor in an audit. Instead, it offers more ways to verify findings in more complex and larger environments. If we look at the current trends in information technology, it is evident that cloud-based solutions have become the go-to solution for many organizations. Assessing these environments can be challenging and any automated tools to help gather and analyse vast amounts of information are welcomed.

Additionally, a CAB can extend its service offering with continuous auditing services and improve audit effectiveness by implementing new innovations and technologies to the continuous auditing process. In the context of EUCS and MEDINA, a CAB could offer the following:

- EUCS and MEDINA certification training:
  - General, publicly available training aiming to aid understanding the concepts of EUCS, MEDINA and associated certification process.

- Certification audits for assurance level Substantial:
  - Certification audits according to EUCS specification.

- Certification audits for assurance level High with MEDINA:
  - Certification audits with continuous auditing services utilizing MEDINA.
  - Different MEDINA components used by the auditor.

MEDINA and EUCS will provide significant business opportunities in the future. The demand for better assurance in cloud environments is already there, and MEDINA helps to provide supply to that demand.

### Changes in audit methods and audit process

The addition of continuous auditing naturally means that the audit process is changed. The traditional approach to auditing, simplified drastically, is to review documentation, interview suitable persons and verify findings by conducting additional tests, such as process observations, sample reviews or technical tests. By utilizing continuous auditing, the last step, verification of results, can be done based on the results collected by automated tools.

The undeniable truth is that continuous auditing will benefit the auditor massively by doing much of the work for the auditor. The change is similar to what industrialization did to manufacturing: human touch is lost in the grass-root level of the process but is better utilized in a more productive role. Continuous auditing itself does not mean that the role of a CAB would be to just check measurement results and grant a certificate, but rather oversee that the whole automation process is implemented and functioning as intended in each auditee environment. Thus, in an EUCS audit as part of the initial audit covered in section "From point-in-time audits to Continuous Auditing" the CAB will have to do a traditional (manual) assessment of the current situation based on the selected EUCS assurance level, and in level high the CAB has to evaluate the implementation of continuous monitoring components and confirm that the measurement results cover the scope of the audit and provide truthful results. Practically speaking, this would add a step to the audit to validate the configuration of the continuous monitoring tool implementation. However, this added time will be gained later when the evidence is assessed automatically.

Automated evidence gathering allows larger samples over longer periods of time to be used in an audit. On the other hand, some manual work is still required but with a standardized design of metrics this gap between automated monitoring and manual auditing methods can be narrowed down significantly. Finally, while certain parts of requirements can be assessed automatically by using measurable metrics, it does not mean that assessing all requirements in compliance frameworks can be fully automated or that all results could be approved as such and thus a 100% automation coverage will not be feasible. As an example, automated natural language processing tools can be used to verify that certain processes are documented in policies, but the verification of these processes might require human interviews or manual process observations.

The initial certification audit of an organization will most likely require more work than before. The implementation of MEDINA has to be verified in addition to the initial certification audit conducted by the auditor. However, this pays off in the surveillance audit which in turn does not require as much work as before, assuming that the results gained through continuous auditing can be utilized.

### MEDINA validation to trust the evidence

Auditors need verification to trust the evidence collected by MEDINA. To trust the evidence, the auditor must accept the use of MEDINA components and trust the results. Why could this be a challenge? There is always a trust component involved when an auditor is reviewing evidence from an information system – has it been tampered with, is the evidence representing a truthful image of the circumstances, etc. As an added component to the audit process, in addition to validating the evidence in relation to the requirements, the auditor should also validate the implementation of the MEDINA framework in order to have assurance that the framework provides suitable evidence. At least the following items should be considered:

- MEDINA is implemented throughout the scope and all applicable assets are being audited.

- All applicable requirements are being audited in all applicable assets.
  - The selected metrics are correct, suitable and meet the intent of the requirements.

- The metrics are correct, and measurements show truthful results.

- The evidence is protected from tampering and any attempt to alter the data can be identified.
  - MEDINA explores the leveraging of Blockchain and other innovative solutions to ensure integrity and accountability. For further reading see MEDINA deliverable D4.3 ^17^

- The audit trail is long enough to validate events in a specified time frame, e.g., since the last audit.

Based on the analysis of a Proof-Of-Concept ^18^ on the metrics and framework created in the MEDINA project, it is possible to implement continuous monitoring which fulfils the intent of the written requirements by using automated evidence collection and analysis. This provides high expectations for the future, and it is likely that we will see a change in how audits are conducted and how the certification is managed. However, the implementation of the metrics must be evaluated case-by-case as each environment and scope is different in each audit. Like in many cases, industry best practices and guidance of governing bodies will eventually steer the notion of continuous auditing towards a standardized way. New metrics and ways to implement metrics will need constant development to suit the needs of different emerging cloud technologies and innovative solutions to ensure that the MEDINA framework stays up-to-date.

### Challenges in continuous auditing and MEDINA

The maturity of MEDINA, and in broader context continuous auditing, is still low which highlights a few obvious challenges. Some of them are already presented in the earlier chapters.

Audits rarely provide 100 % certainty and coverage, and MEDINA does not offer a silver bullet to the issue. MEDINA continuous monitoring is applied to a subset of EUCS requirements (level: high) and therefore continuous auditing does not cover each and every requirement. The approach is justified because the point is to provide better assurance in certain requirements in the high-category, but this limitation has to be kept in mind to prevent a false impression of assuming that continuous monitoring/auditing would provide 100 % coverage of all requirements.

The importance of correct, precise, and well implemented metrics becomes a critical element in continuous auditing. The metrics need to be quite low-level for computers to process them (binary true/false/equals to, value range, less/more than operations, etc.). More metrics are needed to achieve a higher degree of trust in the results. A competent auditor knows when to ask clarifying and more detailed questions to establish whether a solution is conforming to a requirement. In a similar fashion the metrics need to be continually improved to have the ability to use even more metrics to monitor each requirement.

A fundamental property of continuous auditing so far has been the focus on technical controls. While organizational controls can be automatically monitored to some extent, to date not a single automation system can identify all user errors or violations of processes and policies. Some manual inspection by the auditor will always be necessary. Perhaps in the future the use of AI and other innovative technologies will narrow down the gap, but for now continuous auditing cannot provide feasible solutions to all audit needs.

Continuous cloud auditing also includes a supply chain challenge. Modern cloud services use interfaces and connections to multiple internal and external services, and monitoring all these interfaces may be a challenging task. As long as all parts of the supply chain are not certified or at least audited, compliance with requirements in all locations where data is processed, transmitted or stored is uncertain. Finally, from a competence perspective, the adaption of MEDINA and continuous auditing is significant for auditors but also for all other stakeholders. It takes time and effort to get the framework applicable to a real-world cloud environment. 

### From continuous auditing to continuous certification

The term continuous certification currently exists as a concept and is not widely adopted in certification schemes. Why so? Simply because it would require continuous auditing which itself is not a mature concept. Ideally, once the maturity of continuous auditing is high enough, it will lead to continuous certification where the status of the certificate is automatically monitored and updated based on the continuous assessment results. The challenge in the leap from continuous auditing to continuous certification is the fact that scheme owners have a major role in defining the boundaries on how this will be done. MEDINA proposes tools for managing the certificate life cycle ^17^, but the actual decision-making process is heavily dependent on EUCS and other certification schemes. There could be multiple implementation methods for continuous certification varying from auditee-implemented evidence storage solutions with auditor access to sophisticated auditor-implemented SOC-type (Security Operation Center) monitoring solutions. However, the approved solutions are to be chosen by the scheme owners and industries since continuous certification will change the whole ecosystem of how certificate registries work. There are still some challenges to be solved such as:

- What is the process for changing the status of certificates?

- How are findings categorized as major and minor non-conformities automatically?

- What are the different thresholds when multiple minor non-conformities start to affect certification?
  - What sort of timeline is acceptable for corrective actions before the auditor/automated system must react to the situation?

- What are the criteria for certificate suspension in a tool-assisted, automated decision making?
- Is the certificate suspended automatically after a major finding or after the auditor’s analysis?

- How is the certificate status logged throughout the cycle?
  - Is only the current state of the certificate available for the public, or is a log of certificate statuses available?
  - What happens if a major nonconformity causes an incident which has long-term effects, e.g., a data loss prevention control fails and data is lost to an unauthorized party?
  - How is the certificate status updated because simply fixing the control does not revert the original data loss?

The optimal solution could be that all significant findings leading possibly to certificate suspension would be evaluated by the auditor during a grace period and the evidence of all non-conformities would be saved throughout the certification life-cycle. By this way the probability of false positive findings affecting certification is minimized. As a downside, frequent auditor involvement might cause extensive time periods of uncertain certificate status and added work if non-conformities are frequent. Risk management is a major component in continuous certification to assess findings and their effects on certification. For example, a failure in a security control in a low-priority system versus a critical system would have a very different outcome. The failure is a nonconformity whatsoever, but the risk involved might be a deciding factor whether the nonconformity is a major or minor nonconformity. MEDINA includes a risk assessment component which can be utilized when assessing the risk associated to each asset and each finding.

## Further considerations

This section discusses further considerations which could be potential targets for future research and most definitely will help the market adoption of continuous auditing and continuous certification.

### Standardization & regulation

For MEDINA, and moreover in a larger context, continuous auditing and certification to become an industry best practice, it needs systematic standardization activities within the scope of MEDINA but most importantly by the information security certification industry, scheme owners and regulators. Certification schemes usually divide responsibilities and duties between different actors in order to avoid conflicts of interest. The parties owning and developing a certification scheme rarely do audits themselves but focus on maintaining the standard. Vice versa, this also means that auditors cannot start to offer continuous audit and certification services unless the standards, tools, market demand and standardized processes are in place. To make continuous audit and certification a norm and industry best practice, a joint effort consisting of standard development and standardization, regulation by authorities, market demand from cloud customers and strong CAB involvement is required.

Some of the CSPs will voluntarily start implementing continuous auditing such as MEDINA to gain a competitive advantage. They see the added value it creates in terms of business and in security. Unfortunately, all organizations will not be like that, and this emphasizes the need for regulation to encourage and enforce the change. Too heavy regulation will not be a feasible solution but mandatory continuous auditing in specified business sectors for certain critical environments could be an excellent starting point.

### Trust through transparency

To gain the trust of cloud service providers, cloud customers and auditors, MEDINA needs to be transparent. A black box producing results without describing how results were achieved will not satisfy any auditor. Moreover, no organization will install a component to any business-critical environment if its trustworthiness from a security perspective and evidence trustworthiness perspective cannot be verified. When auditors have the possibility to understand and verify the trustworthiness of MEDINA components and the specific implementation in each auditee’s environment, the results can be trusted. Trustworthiness is not only a concern for auditors but also for CSPs. There is no point in using a system if the trustworthiness cannot be verified. Considering that the tools aim to help understand the security status of an environment, false information is not beneficial to anyone.

Eventually, the solution could be that the MEDINA components are either provided as open-source and/or certified with a relevant certification, but currently there are no feasible product certification solutions. Additionally, certification of these tools does not guarantee reliable results since each installation is always depending on the environment and implementation where it is installed. While a certification would guarantee a certain security baseline, the implementation of the MEDINA tools in the target environment still has a significant impact on the results. The auditor must verify the trustworthiness of the configuration in each case separately and the level of detail this requires might vary. A widely adopted “industry accepted” tool might be rather simple to verify but a custom-made solution could require specialized skills to understand. Like in many cases, the acceptance criteria for the tools will likely develop as the tools become more common.

## Conclusions

This paper has provided a brief introduction to MEDINA and its proposed solution to answer a challenge faced by many organizations from the perspective of a security auditor. By achieving continuous auditing, the cloud computing industry can significantly increase the level of trust in a single audit, but eventually in the certificate itself. For auditors MEDINA offers a fantastic opportunity to add another audit method to be used in an audit and further increase their trust in the audit results.

The involvement of the automation component in the audit will have changes in all levels of the audit process starting from the verification of the used tools to verifying pieces of evidence. One has to ensure that continuous auditing is implemented correctly to have trust in the results. To achieve that, transparency of the framework, tools and customer implementation is a key factor. Additionally, adjusting the audit processes will need some time and guidance. However, when done correctly, continuous auditing allows much larger sample sizes from a much longer time period when time can be focused on finding non-conformities from results instead of manual assessments based on the auditor’s sample. Continuous certification is the next step from continuous auditing. In order for it to work and become an industry best practice, it requires continuous auditing to be a mature and widespread concept. In addition to that, scheme owners need to define the requirements and guidelines for automated certification life cycle management. MEDINA proposes solutions to all of these challenges, but the scheme owners are the authority to decide the proper approach.

## Ethics and consent

Ethical approval and consent were not required.

## Data Availability

No data are associated with this article.

## Footnotes

^1^ https://medina-project.eu/public-deliverables/
