# Supplementary material for: Continuous Auditing and Continuous Certification of Cloud Services in MEDINA – Security Auditor’s View
Source: Open Res Eur. 2024 May 1;3:208. Originally published 2023 Nov 23. [Version 2] doi: 10.12688/openreseurope.16703.2 (PMC11196927; doi:10.12688/openreseurope.16703.2)
Supplement: Peer review history

# Reviewer report: Reviewer response for version 2

**DOI:** 10.21956/openreseurope.18738.r40049

**Published:** 2024-06-24

**Authors:** Henrique Santos

**Affiliations:** 1 Department of Information Systems, Universidade do Minho, Braga, Braga, Portugal

The paper seems more consolidated to me, and after the adjustments made, I understand that it is ready to be approved. Congratulations to the authors on the work carried out. I hope that the paper helps to motivate more researchers to develop work in this area, especially in an industrial control environment.

Where applicable, are recommendations and next steps explained clearly for others to follow? (Please consider whether others in the research community would be able to implement guidelines or recommendations and/or constructively engage in the debate)

Not applicable

Does the article adequately reference differing views and opinions?

No

Are all factual statements correct, and are statements and arguments made adequately supported by citations?

Yes

Is the rationale for the Open Letter provided in sufficient detail? (Please consider whether existing challenges in the field are outlined clearly and whether the purpose of the letter is explained)

Partly

Is the Open Letter written in accessible language? (Please consider whether all subject-specific terms, concepts and abbreviations are explained)

Yes

Reviewer Expertise:

Cybersecurity; Computer Engineering; Information Systems

I confirm that I have read this submission and believe that I have an appropriate level of expertise to confirm that it is of an acceptable scientific standard.

# Reviewer report: Reviewer response for version 2

**DOI:** 10.21956/openreseurope.18738.r40048

**Published:** 2024-05-17

**Authors:** Johan David Michels

**Affiliations:** 1 Queen Mary University of London, London, England, UK

The revisions address my comments. Many thanks to the authors and congratulations on a well-written piece discussing an important topic. I hope it will prove useful to many readers.

Where applicable, are recommendations and next steps explained clearly for others to follow? (Please consider whether others in the research community would be able to implement guidelines or recommendations and/or constructively engage in the debate)

Yes

Does the article adequately reference differing views and opinions?

No

Are all factual statements correct, and are statements and arguments made adequately supported by citations?

Yes

Is the rationale for the Open Letter provided in sufficient detail? (Please consider whether existing challenges in the field are outlined clearly and whether the purpose of the letter is explained)

Yes

Is the Open Letter written in accessible language? (Please consider whether all subject-specific terms, concepts and abbreviations are explained)

Yes

Reviewer Expertise:

Cybersecurity regulation; cloud computing law; data protection law; commercial law studies

I confirm that I have read this submission and believe that I have an appropriate level of expertise to confirm that it is of an acceptable scientific standard.

# Reviewer report: Reviewer response for version 1

**DOI:** 10.21956/openreseurope.18041.r36549

**Published:** 2024-03-12

**Authors:** Johan David Michels

**Affiliations:** 1 Queen Mary University of London, London, England, UK

I commend the authors for writing a well-written, accessible open letter introducing the notion of continuous auditing and certification. The topic is of interest to those involved in the regulation of cybersecurity, including through certification schemes. The paper sets out clearly the differences between (1) point-in-time audits; (2) continuous auditing; and (3) continuous certification. Cloud computing, the EUCS, and MEDINA provide a helpful practical context to illustrate these points. I recommend the article for indexing, subject to the below revisions. (As a caveat, I add that my expertise is in cybersecurity regulation, not in computer science or security auditing, so I cannot comment on all the technical aspects of the analysis.)

 First, in terms of style, there are a few minor language errors, which could be easily fixed in copy-editing (e.g. p. 3 "the its risk appetite" and "Digital evidence are", or p.5 "the opportunities [...] is" and "great opportunities both the auditee".) 

 Second, in terms of substance, my main concern is that the paper does not reflect on the possible downsides of a continuous auditing approach. As a result, it comes across as rather one-sided in favour of MEDINA. While continuous auditing has clear benefits over point-in-time audits, the paper could for instance consider whether automated monitoring of metrics can fully capture relevant vulnerabilities. It strikes me that some security measures (e.g. is software up to date; is the firewall configured correctly; is two-factor authentication set up where appropriate) can be better captured through such metrics than others (e.g. do accounts have role-appropriate privileges). For other measures, I'm just not sure (e.g. are any users using default or weak passwords, or re-using compromised passwords from private accounts; how susceptible are users to phishing attacks, etc.). It would be helpful if the paper discussed some of the weaknesses and identified the limits of this approach. A separate question is the extent to which such monitoring could apply to any underlying sub-processors in the cloud provider's supply chain.

 In addition, I think it would be helpful if the paper mentioned a practical example of a metric that lends itself well to continuous auditing. For example, the paper could spell out a security objective; then state how that objective can be achieved through a certain security measure; and then explain how the implementation of that measure can be measured by means of a metric that can be automatically monitored so that continuous auditing can provide a level of assurance. It need only be a single paragraph, but it might help readers who are not versed in security auditing (such as myself) to understand the concept better.

 Finally, I offer an observation, without per se suggesting any revision. On p.7, the authors suggest risk management as a component in continuous certification, with the risk involved being a deciding factor whether a non-conformity is major or minor, which in turn helps determine consequences in terms of either (1) correcting the non-conformity during a grace period; or (2) suspending certificate status. I note that such a risk-based approach would be in line with the risk-based approach to incident notification under the GDPR Art. 33-34 and the NISD Art.16. Going further, one might consider continuous auditing to be part of a bigger, layered system of notifications under EU law, in which at the lowest level, a non-conformity is notified to an external auditor for analysis; which in case of a major non-conformity could lead to a CAB notification and possibly a certificate suspension; while a security incident (that is: an actual breach) must be notified to a regulator (under GDPR and/or NSID) for potential investigation and enforcement (including fines). Just a thought.

Where applicable, are recommendations and next steps explained clearly for others to follow? (Please consider whether others in the research community would be able to implement guidelines or recommendations and/or constructively engage in the debate)

Yes

Does the article adequately reference differing views and opinions?

No

Are all factual statements correct, and are statements and arguments made adequately supported by citations?

Yes

Is the rationale for the Open Letter provided in sufficient detail? (Please consider whether existing challenges in the field are outlined clearly and whether the purpose of the letter is explained)

Yes

Is the Open Letter written in accessible language? (Please consider whether all subject-specific terms, concepts and abbreviations are explained)

Yes

Reviewer Expertise:

Cybersecurity regulation; cloud computing law; data protection law; commercial law studies

I confirm that I have read this submission and believe that I have an appropriate level of expertise to confirm that it is of an acceptable scientific standard, however I have significant reservations, as outlined above.

# Response

**Published:** 2024-04-08

**Authors:** Cristina Martinez

**Affiliations:** DIGITAL, Fundacion Tecnalia Research & Innovation, San Sebastián, Basque Country, Spain

Dear Johan David, Thank you very much for reviewing our article and for your helpful comments. We have addressed them in version v2 of the article. You can see our responses below: First, in terms of style, there are a few minor language errors, which could be easily fixed in copy-editing (e.g. p. 3 "the its risk appetite" and "Digital evidence are", or p.5 "the opportunities [...] is" and "great opportunities both the auditee".) [MMC1] 

Second, in terms of substance, my main concern is that the paper does not reflect on the possible downsides of a continuous auditing approach. As a result, it comes across as rather one-sided in favour of MEDINA. While continuous auditing has clear benefits over point-in-time audits, the paper could for instance consider whether automated monitoring of metrics can fully capture relevant vulnerabilities. It strikes me that some security measures (e.g. is software up to date; is the firewall configured correctly; is two-factor authentication set up where appropriate) can be better captured through such metrics than others (e.g. do accounts have role-appropriate privileges). For other measures, I'm just not sure (e.g. are any users using default or weak passwords, or re-using compromised passwords from private accounts; how susceptible are users to phishing attacks, etc.). It would be helpful if the paper discussed some of the weaknesses and identified the limits of this approach. [MMC2] A separate question is the extent to which such monitoring could apply to any underlying sub-processors in the cloud provider's supply chain [TS4] 

In addition, I think it would be helpful if the paper mentioned a practical example of a metric that lends itself well to continuous auditing. For example, the paper could spell out a security objective; then state how that objective can be achieved through a certain security measure; and then explain how the implementation of that measure can be measured by means of a metric that can be automatically monitored so that continuous auditing can provide a level of assurance. It need only be a single paragraph, but it might help readers who are not versed in security auditing (such as myself) to understand the concept better. [MMC5] 

Finally, I offer an observation, without per se suggesting any revision. On p.7, the authors suggest risk management as a component in continuous certification, with the risk involved being a deciding factor whether a non-conformity is major or minor, which in turn helps determine consequences in terms of either (1) correcting the non-conformity during a grace period; or (2) suspending certificate status. I note that such a risk-based approach would be in line with the risk-based approach to incident notification under the GDPR Art. 33-34 and the NISD Art.16. Going further, one might consider continuous auditing to be part of a bigger, layered system of notifications under EU law, in which at the lowest level, a non-conformity is notified to an external auditor for analysis; which in case of a major non-conformity could lead to a CAB notification and possibly a certificate suspension; while a security incident (that is: an actual breach) must be notified to a regulator (under GDPR and/or NSID) for potential investigation and enforcement (including fines). Just a thought. [TS7] [MMC1]Language errors have been corrected [MMC2]This suggestion has been addressed in the new section entitled “Challenges in continuous auditing and MEDINA” [TS4]Added a chapter called “ **Challenges in continuous auditing and MEDINA ** “ [MMC5]An example has been onroduced in the section entitled "MEDINA framework" [TS7]I agree with the reviewer, but this issue is something to be decided by the EU and scheme owners. I like the idea that the notification system would be built in to the system but it is out of the scope of this project.

# Reviewer report: Reviewer response for version 1

**DOI:** 10.21956/openreseurope.18041.r36543

**Published:** 2024-01-02

**Authors:** Henrique Santos

**Affiliations:** 1 Department of Information Systems, Universidade do Minho, Braga, Braga, Portugal

The paper addresses a very pertinent question that may be of interest to the entire cybersecurity research community, particularly management and certification. It doesn't follow a scientific structure or a scientific method, but it illustrates a model that could be useful.

 The use of bibliographical references does not follow a standard format, but this is an aspect that the editors could consider.

 The authors can improve the paper with the following changes:

 1. The title should explicitly mention the application context: Cloud Computing

 2. Continuous monitoring and certification is not exactly a new topic. We would therefore expect some references to this subject and how it has been approached in other contexts.

 3. One of the most complex problems in OT certification (continuous or otherwise) is the correct choice of metrics. The paper states that the metrics are derived from the security requirements/objectives defined in the EUCS, pointing to another project deliverable. However, a description, even if minimal, of how the metrics are identified would be desirable. They go on to say that they use a dedicated language to facilitate the automatic identification of metrics, but the way they do it could be clearer.

 4. It is not expected that all security objectives, especially those of an organisational and operational nature, will benefit from a continuous certification scheme. It would be interesting to have a section reflecting on the impact of what can/should be continuously audited in a full cybersecurity audit programme (even if restricted to CSPs).

Where applicable, are recommendations and next steps explained clearly for others to follow? (Please consider whether others in the research community would be able to implement guidelines or recommendations and/or constructively engage in the debate)

Not applicable

Does the article adequately reference differing views and opinions?

No

Are all factual statements correct, and are statements and arguments made adequately supported by citations?

Yes

Is the rationale for the Open Letter provided in sufficient detail? (Please consider whether existing challenges in the field are outlined clearly and whether the purpose of the letter is explained)

Partly

Is the Open Letter written in accessible language? (Please consider whether all subject-specific terms, concepts and abbreviations are explained)

Yes

Reviewer Expertise:

Cybersecurity; Computer Engineering; Information Systems

I confirm that I have read this submission and believe that I have an appropriate level of expertise to confirm that it is of an acceptable scientific standard, however I have significant reservations, as outlined above.

# Response

**Published:** 2024-04-08

**Authors:** Cristina Martinez

**Affiliations:** DIGITAL, Fundacion Tecnalia Research & Innovation, San Sebastián, Basque Country, Spain

Dear Henrique, Thank you very much for reviewing our article and for your helpful comments. We have addressed them in version v2 of the article. You can see our responses below: The use of bibliographical references does not follow a standard format, but this is an aspect that the editors could consider [MMC1] . The authors can improve the paper with the following changes: 1. The title should explicitly mention the application context: Cloud Computing. [MMC2] 2. Continuous monitoring and certification is not exactly a new topic. We would therefore expect some references to this subject and how it has been approached in other contexts. [MMC3]  3. One of the most complex problems in OT certification (continuous or otherwise) is the correct choice of metrics. The paper states that the metrics are derived from the security requirements/objectives defined in the EUCS, pointing to another project deliverable. However, a description, even if minimal, of how the metrics are identified would be desirable. They go on to say that they use a dedicated language to facilitate the automatic identification of metrics, but the way they do it could be clearer. [MMC4] 4. It is not expected that all security objectives, especially those of an organisational and operational nature, will benefit from a continuous certification scheme. It would be interesting to have a section reflecting on the impact of what can/should be continuously audited in a full cybersecurity audit programme (even if restricted to CSPs). [MMC5] [MMC1]We have used the format of references defined in the ORE template for Overleaf (APA standard) [MMC2]The tltle has been changed as follows: " **Continuous Auditing and Continuous Certification of Cloud Services in MEDINA – Security Auditor’s View "** [MMC3]We have elaborated on this suggestion at the end of the "Introduction" section. In addition, 3 references to papers have been added. [MMC4]A sentence about how metrics have been elicited has been included in the section "MEDINA framework" [MMC5]The "Introduction" section of the paper explains that the security objectives that can be continously monitored are precisely identified in the  EUCS, and belong to the high assurance level. The focus ol MEDINA is on these high level requirements that are suitable for continuos automated monitoring. Organizational objectives can be also continously monitored in MEDINA, but the periodicity defined for them is much higher than for operational controls. We have added a parapraph in section "MEDINA framework" to clarify it. Best regards, Crsitina Martinez
